# LPS-induced serum TNF production and lethality in mice: effect of L-carnitine and some acyl-derivatives

**DOI:** 10.1155/S0962935193000754

**Published:** 1993

**Authors:** Vito Ruggiero, Claudio M. D'Urso, Claudio Albertoni, Silvia Campo, Piero Foresta, Edoardo Arrigoni Martelli

**Affiliations:** Sigma Tau Ind. Farm., Riun. SpA, Research and Development Department of Microbiology and Immunology Via Pontina Km. 30 Pomezia 400, 00040 Italy

## Abstract

The effect of L-carnitine and some of its acyl derivatives on serum TNF production and lethality in a murine experimental endotoxin shock model was investigated. In some instances, serum IL-6 production was also evaluated. In this experimental model, C57BL/6 mice received 30 mg/kg LPS (E. cell 055:B5) injected intraperitoneally, while L-carnitine and its derivatives were administered according to different schedules. Serum levels of TNF and IL-6 were evaluated 1 h following LPS injection. The treated animals were also monitored daily for differences in body temperature, feeding, and survival for 10 days after LPS injection. Although some derivatives were able to significantly affect TNF production, the marked decrease in serum TNF levels of LPS-treated mice was not paralleled by a substantial increase in survival.


Research Paper

Mediators of Inflammation 2, S43–S50 (1993)

### LPS-induced serum TNF production and lethality in mice: effect of L-carnitine and some acyl-derivatives

Vito Ruggiero, Claudio M. D'Urso, Claudio Albertoni, Silvia Campo, Piero Foresta[CA] and Edoardo Arrigoni Martelli

Sigma Tau Ind. Farm., Riun. SpA, Research and Development, Department of Microbiology and Immunology, Via Pontina Km. 30, 400, 00040 Pomezia, Italy

[CA] Corresponding Author

**THE effect of L-carnitine and some of its acyl derivatives on serum TNF production and lethality in a murine experimental endotoxin shock model was investigated. In some instances, serum IL-6 production was also evaluated. In this experimental model, C57BL/6 mice received 30 mg/kg LPS (*E. coli* 055:B5) injected intraperitoneally, while L-carnitine and its derivatives were administered according to different schedules. Serum levels of TNF and IL-6 were evaluated 1 h following LPS injection. The treated animals were also monitored daily for differences in body temperature, feeding, and survival for 10 days after LPS injection. Although some derivatives were able to significantly affect TNF production, the marked decrease in serum TNF levels of LPS-treated mice was not paralleled by a substantial increase in survival.**

**Key words:** Carnitines, Endotoxaemia, Endotoxin shock, Hypothermia, IL-6, LPS, Mice, Septic shock, TNF

### Introduction

Septic shock is a clinical syndrome associated with a high lethality rate, and characterized by various haemodynamic and immuno-biochemical alterations.[1] This disease is primarily caused by Gram-negative bacteria, but it may also be consequent to infections by some Gram-positive bacteria, fungi, and probably parasites and viruses.[2] The pathophysiological effects caused by Gram-negative bacteria have been ascribed to lipopolysaccharide (LPS) a component of the bacterial outer membrane, which has also been termed endotoxin because of its toxic effects.[3] LPS causes septic shock by interacting with various components of the host's immune system, and primarily with macrophages, which in turn release several endogenous mediators that are themselves the ultimate effectors of the disease.[4–6] Recently, the systemic release of large amounts of various cytokines has been associated with fatal outcome in human septic shock.[7,8]

TNF is one of the cytokines considered to play a pivotal role as a mediator of the host's response to LPS.[9] Therefore, blocking or antagonizing TNF in sepsis may have therapeutic potential. Furthermore, making TNF a target for intervention, rather than LPS, may be more advantageous in that TNF has also been found to be involved in the pathogenesis of shock due to Gram-positive bacteria.[10,11] Among the drugs used in giving metabolic support to septic patients, L-carnitine has been found to be a fairly good candidate to ameliorate the host's metabolic response to septic processes.[12–14]

L-Carnitine is a drug that is not only essential for the mitochondrial oxidation of long-chain fatty acids, but also for intercompartmental shuttling of energy substrates as well as elimination of toxic metabolites, and modulation of the free CoA/Acyl-CoA ratio.[15] Although the information concerning the effects of carnitine on cells of the immune system is still scanty, a report showed that L-carnitine effectively inhibited chemiluminescence in PMA-stimulated human PMN leukocytes.[16] This is of some interest in the light of the findings that both chemiluminescence as well as TNF production are phenomena negatively regulated by cyclic nucleotides.[17,18] In this report the authors investigated the effect of L-carnitine and some of its acyl derivatives on endotoxin-induced serum TNF production, lethality, and some physiological responses (feeding and body temperature) in a murine experimental endotoxin shock model.

Experimental endotoxaemia has become a valuable experimental model for septicaemia in laboratory animals. Although this model does not completely reproduce all the features of clinical septic shock, the accumulated evidence shows that it reliably mimics Gram-negative sepsis.[3] Another cytokine, IL-6, has recently been implicated in the pathophysiology of septic shock,[19] and a report has also shown that it may play a role in endotoxin treated mice.[20] In some instances, therefore, the

© 1993 Rapid Communications of Oxford Ltd effects of L-carnitine on serum IL-6 levels in our model of endotoxin shock were also evaluated.

### Materials and Methods

*Animals:* Male C57BL/6 mice were purchased from Iffa-Credo (Lyon, France). Mice (6–7 weeks of age at the time of use) were housed in groups of eight, and were allowed food and water *ad libitum*. They were kept under specific pathogen-free conditions, and were randomly assigned to various treatment groups.

*Drugs and chemicals:* L-Carnitine as well as its derivatives were all synthesized by Sigma-Tau Chemical Laboratories (Pomezia, Italy). LPS (*Escherichia coli* 055:B5) was from Difco (Difco Laboratories, Detroit, MI, USA); actinomycin D-mannitol was from Sigma (Sigma Chemical Laboratories, St Louis, MO, USA). Recombinant murine TNF and recombinant murine IL-6 were both purchased from Genzyme (Boston, MA, USA). All chemicals were dissolved in pyrogen-free physiological saline solution just prior to use. Tritiated thymidine ($^3$H-TdR, 5 Ci/mmol) was from Amersham (Aylesbury, Buckinghamshire, UK).

*Cell lines and tissue culture media:* The TNF-sensitive L929 cell line (a murine fibrosarcoma) was grown in RPMI-1640 medium containing 25 mM HEPES and 2 mM L-glutamine (Biochrom KG, Berlin, Germany) supplemented with 10% heat-inactivated Myoclone foetal calf serum (FCS) (Gibco, Grand Island, NY, USA), and 50 $\mu$g/ml gentamicin (Sigma). The murine hybridoma cell line B9, which is dependent on IL-6 for growth[21] was maintained in the above culture medium with the further addition of $5.0 \times 10^{-5}$ M 2-mercaptoethanol (Sigma) and 10 U/ml recombinant murine interleukin 6 (rmIL-6).

*Endotoxaemia model:* C57BL/6 mice received 30 mg/kg LPS intraperitoneally (i.p.), which had been predetermined to produce approximately 60% lethality. In the majority of experiments, animals were first pre-treated i.p. with L-carnitine derivatives 1 h before LPS injection, and then treated intravenously (i.v.) 10 min following LPS administration. However, other treatment protocols were also adopted, and they are reported in the relevant tables. Some animal physiological responses to endotoxaemia were graded such as food intake, body weight, mobility, amount of ocular exudate, and rectal temperature.

In particular, temperature measurements were taken with an electronic thermometer (model Babuc/M, I.S.I., Milan, Italy) with the appropriate thermistor (probe TM35S). Animal health state was classified as follows: very ill (body temperature below 32°C, absence of mobility, marked body weight loss, abundant ocular exudate); fair (body temperature between 32 and 35°C, poor mobility, small body weight loss, moderate ocular exudate); normal (body temperature above 35°C, normal mobility, normal body weight, absence of ocular exudate). All groups of animals were examined at 24 h intervals after LPS injection to assess survival, and they were followed up to 10 days.

*Serum TNF and IL-6 determinations:* To determine circulating TNF and IL-6 levels, ether-anaesthetized mice were bled by retro-orbital sinus puncture at 1 h following 30 mg/kg LPS injection. Blood was allowed to clot at room temperature, and then centrifuged at 1 000 × *g* for 15 min. The separated serum was stocked and stored at −80°C until assayed.

*TNF bio-assay:* Concentrations of TNF in serum were determined by using the L929 cytotoxicity assay described by Flick and Gifford[22] with minor modifications. Briefly, 100 $\mu$l of L929 cells ($3.2 \times 10^5$ cells/ml) in RPMI-1640 containing 10% FCS were seeded into each well of a flat-bottomed 96-well microtitre plate (Falcon, Becton & Dickinson, Meylan Cedex, France) and incubated overnight at 37°C in a humidified atmosphere of 5% $CO_2$. After incubation, spent medium was discarded and two-fold serial dilutions (carried out in RPMI-1640 1% FCS) of serum samples were added to the cells in the presence of actinomycin D-mannitol at a final concentration of 1 $\mu$g/ml. The L929 cell cultures were then further incubated for 18 h. Following incubation, the medium was discarded, and the plates were first washed twice with 0.9% NaCl and then stained for 15 min at room temperature with 0.5% (w/v) Crystal Violet in 20% ethanol and 8% formaldehyde. After discarding the stain, the wells were gently washed in running tap water; the dye which had been taken up was eluted with 33% acetic acid and the relative absorbance at 540 nm was measured with a Multiskan MCC/340 ELISA reader (Flow Laboratories, Mclean, VA, USA). TNF activity is expressed in units/ml, and is defined as the reciprocal of the dilution necessary to cause death of 50% of the actinomycin-treated control cells. Final TNF concentrations for each sample were calculated by interpolating the values of four to six sample dilutions.

*IL-6 bio-assay:* Levels of IL-6 in serum were measured by its ability to induce proliferation of the murine B-cell hybridoma line B9.[21] B9 cells were washed twice prior to use in the assay in order to remove the exogenous rmIL-6 added to sustain their growth.

Briefly, two-fold serial dilutions of thawed sera in RPMI-1640 supplemented with 10% FCS,

$5.0 \times 10^{-5}$ M 2-mercaptoethanol were added to flat-bottomed 96-well microtitre plates containing $5.0 \times 10^3$ cells per well. Cells were then incubated for 96 h at 37°C in a humidified atmosphere of 5% $CO_2$, and pulsed with 0.5 $\mu$Ci/well of $^3$H-TdR during the last 18 h of incubation. Afterwards, the cells were harvested onto glass fibre filtermats (Pharmacia, Turku, Finland) by using an automated cell harvester (Pharmacia), and $^3$H-TdR incorporation (an index of cell proliferation) was assessed by a $\beta$-plate counter (Pharmacia). IL-6 activity is expressed in B9 hybridoma growth units per ml, and one unit is defined as the reciprocal of the sample dilution required to produce one half-maximal proliferation. Values for each sample were calculated by interpolating the sample cpm of four to six dilutions by means of linear regression analysis.

*Statistical analysis:* Statistical analysis of TNF and IL-6 values was performed by using the Student's *t*-test, while survival data were assessed by Fisher's exact test.

### Results

*Physiological responses to endotoxin:* LPS intraperitoneal injection in C57BL/6 mice (30 mg/kg body weight) provoked a considerable serum TNF release (Fig. 1). Systemic TNF secretion started as early as 0.5 h after LPS administration reaching a peak at 1 h, and then declined to undetectable levels within the next 4 h. Twenty-four hours following LPS challenge, animals exhibited decreased body weights paralleled by concomitant hypothermia, which lasted up to 3 days before they started to recover (Fig. 2). Furthermore, the animals given endotoxin showed an abundant ocular exudate, a dramatic decrease in food intake as well as a loss of mobility and, when touched, a comatose–dazed behaviour.

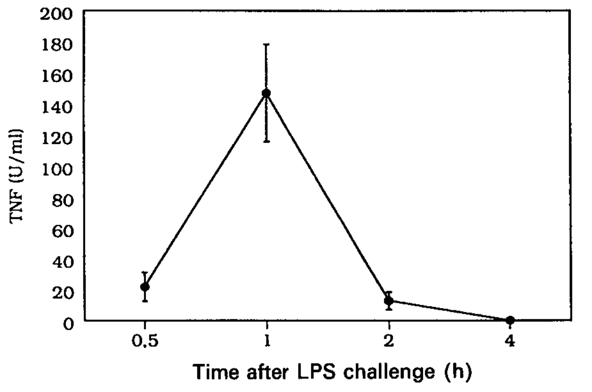

FIG.1. Time course of serum TNF concentration after i.p. administration of 30 mg/kg LPS to C57BL/6 mice. TNF was determined as detailed in Materials and Methods. Values are mean (four animals per group) ± standard deviation.

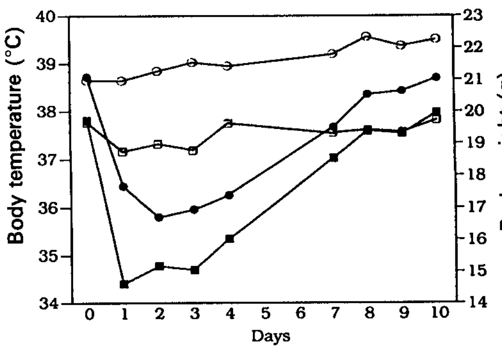

FIG.2. Variations in body temperature (squares) and body weight (circles) in control (open symbols) and LPS-challenged mice (closed symbols). Animals were checked up to 10 days after LPS administration at day 0 (30 mg/kg, i.p.).

*L-carnitine and one derivative:* L-Carnitine (ST 198) caused a marked decrease in serum TNF levels and, at the same time, induced an increased serum IL-6 release. The observed variation in TNF and IL-6 levels was statistically significant ($p < 0.05$), and was confirmed by a second experiment performed according to the same experimental protocol. Although L-carnitine significantly affected serum TNF and IL-6 release, it became clear that, at least at the doses employed in our protocol, it was not able to confer a significant protection upon the animals. In fact, only a small improvement in survival was observed, namely ten survivors in the ST 198-treated group *vs.* eight survivors in the LPS control. Similar results were obtained even with a protocol based on repeated L-carnitine administrations. The ST 784 derivative exerted similar effects both on serum TNF levels and survival, but, at variance with L-carnitine, was unable to modulate IL-6 release (Table 1). Although the survival was not significantly increased after treatment with ST 198 and ST 784, it was found that both compounds markedly improved the health conditions of LPS-challenged mice as evaluated by food intake. The former compound, in particular, appeared to be more active in being able to induce a three-fold increase of food consumption with respect to the LPS control (Table 2).

*Acetyl-L-carnitine and three derivatives:* At the doses utilized throughout the experiments, neither acetyl-L-carnitine (ST 200) nor its derivatives were able to significantly modulate serum TNF release and survival, except ST 1025 which, although unable to affect the animal survival, was still capable of inducing a significant reduction ($p < 0.001$; $p < 0.01$) in TNF levels in two different experiments. The lack of effects of ST 200 and ST 1025 on animal survival was also confirmed when both compounds were administered using the protocol based on multiple administrations. Interestingly, all

**Table 1.** Effects of L-carnitine (ST 198) and its derivative (ST 784) on both serum TNF and IL-6 production, and lethality of mice challenged with LPS

| | | TNF | | | IL-6 | | | Lethality |
|---|---|---|---|---|---|---|---|---|
| Compound | Treatment | % variation | No. of mice | *p* | % variation | No. of mice | *p* | Dead/total |
| ST 198 | *1* (50 mg/kg) | −57 | 8 | <0.05 | +124 | 8 | <0.05 | 14/24 (16/24) |
| | *1* (50 mg/kg) | −64 | 8 | <0.05 | +39 | 8 | <0.05 | |
| | *2* (50 mg/kg) | – | – | – | – | – | – | 6/10 (8/10) |
| ST 784 | *1* (50 mg/kg) | −36 | 24 | <0.02 | +19 | 8 | n.s. | 9/16 (11/16) |
| | *2* (50 mg/kg) | – | – | – | – | – | – | 7/10 (8/10) |

Treatments were performed by injecting LPS 30 mg/kg i.p. at time zero and administering the compounds, at the dose indicated, as follows: *1*) −60 min i.p. and +10 min i.v., *2*) from day −1 to day +3 i.p. twice a day (once at day 0). Statistical significance (*p*) was evaluated by the Student's *t*-test. Lethality of the LPS-treated group is indicated in brackets. ST 198 = L-carnitine inner salt; ST 784 = L-carnitine chloride ester with β-hydroxybutyric acid.

**Table 2.** Health conditions, evaluated by food intake of LPS-challenged mice following treatment with ST 198 and ST 784

| Compound | Food consumed (g) | Cumulative survival (d) | Food consumed per animal per day of survival |
|---|---|---|---|
| Untreated control | 311 | 100 | 3.11 |
| LPS control | 51 | 40 | 1.28 |
| ST 198 | 158 | 53 | 2.98 |
| ST 784 | 105 | 49 | 2.14 |

Mice (ten animals per group) were treated with ST 198 (50 mg/kg, i.p.) and with ST 784 (50 mg/kg, i.p.), twice daily but once at day 0, starting from day −1 to day +3, with respect to the LPS challenge (day 0). Control groups were either injected with 30 mg/kg LPS (LPS control) or treated twice a day with saline from day −1 through day +3 (untreated control). Animals were followed up to 10 d after LPS injection. Food consumed during this time as well as cumulative survival (referred to as the sum of the days of survival of each single mouse within a group) were evaluated. The ratio of the former and the latter parameter yields the food consumed per animal per day of survival (right-end column).

the compounds belonging to this class caused a significant increase in serum IL-6 release (Table 3).

*Propionyl-L-carnitine and three derivatives:* Although propionyl-L-carnitine (ST 261) and its derivatives were all able to decrease serum TNF levels, their effect was not statistically significant. The only exception was ST 780, which lowered ($p < 0.02$) serum TNF release when the treatment schedule included an i.v. administration. No significant changes in serum IL-6 levels were observed in LPS-treated mice following administration with ST 261 and ST 780. All compounds belonging to this class were unable to reduce the LPS-induced lethality, at least at the doses utilized in the experiments (Table 4).

*Isobutyryl-L-carnitine and two derivatives:* Even though the parent compound (ST 284) was not able to influence serum TNF release, its derivatives ST 730

**Table 3.** Effects of acetyl-L-carnitine chloride (ST 200) and its derivatives (ST 857, ST 943, ST 1025) on both serum TNF and IL-6 production, and lethality of mice challenged with LPS

| | | TNF | | | IL-6 | | | Lethality |
|---|---|---|---|---|---|---|---|---|
| Compound | Treatment | % variation | No. of mice | *p* | % variation | No. of mice | *p* | Dead/total |
| ST 200 | *1* (50 mg/kg) | −26 | 8 | n.s. | +61 | 8 | <0.01 | 9/16 (10/16) |
| | *1* (50 mg/kg) | −52 | 8 | n.s. | +20 | 8 | <0.02 | |
| | *2* (50 mg/kg) | – | – | – | – | – | – | 7/10 (8/10) |
| ST 857 | *1* (50 mg/kg) | −11 | 16 | n.s. | +45 | 8 | <0.01 | 10/16 (9/16) |
| ST 943 | *1* (50 mg/kg) | +24 | 8 | n.s. | +105 | 8 | <0.001 | 13/16 (10/16) |
| | *1* (50 mg/kg) | +5 | 16 | n.s. | | | | |
| ST 1025 | *1* (15 mg/kg) | −60 | 16 | <0.001 | +73 | 8 | <0.001 | 11/16 (10/16) |
| | *1* (15 mg/kg) | −74 | 8 | <0.01 | | | | |
| | *2* (15 mg/kg) | – | – | – | – | – | – | 7/10 (8/10) |

Treatments were performed by injecting LPS 30 mg/kg i.p. at time zero and administering the compounds, at the dose indicated, as follows: *1*) −60 min i.p. and +10 min i.v., *2*) from day −1 to day +3 i.p. twice a day (once at day 0). Statistical significance (*p*) was evaluated by the Student's *t*-test. Lethality of the LPS-treated group is indicated in brackets. ST 200 = acetyl-L-carnitine chloride; ST 857 = acetyl-L-carnitine chloride amide with arginine hydrochloride; ST 943 = acetyl-L-carnitine tartrate acid ester with γ-hydroxybutyric acid; ST 1025 = acetyl-L-carnitine amide with citrulline.

**Table 4.** Effects of propionyl-L-carnitine chloride (ST 261) and its derivatives (ST 780, ST 880, ST 921) on both serum TNF and IL-6 production, and lethality of mice challenged with LPS

| | | TNF | | | IL-6 | | | Lethality |
|---|---|---|---|---|---|---|---|---|
| Compound | Treatment | % variation | No. of mice | *p* | % variation | No. of mice | *p* | Dead/total |
| ST 261 | *1* (50 mg/kg) | −37 | 8 | n.s. | +1 | 8 | n.s. | 5/8 (6/7) |
| | *1* (50 mg/kg) | −48 | 8 | n.s. | −2 | 8 | n.s. | |
| ST 780 | *1* (50 mg/kg) | −62 | 8 | <0.02 | +8 | 8 | n.s. | 3/8 (1/8) |
| | *3* (50 mg/kg) | −30 | 5 | n.s. | +6 | 5 | n.s. | |
| | *2* (50 mg/kg) | – | – | – | – | – | – | 5/8 (5/8) |
| ST 880 | *1* (50 mg/kg) | −51 | 8 | n.s. | | | | 6/7 (5/7) |
| ST 921 | *1* (50 mg/kg) | −47 | 8 | n.s. | | | | 6/8 (6/7) |

Treatments were performed by injecting LPS 30 mg/kg i.p. at time zero and administering the compounds, at the dose indicated, as follows: *1*) −60 min i.p. and +10 min i.v., *2*) from day −1 to day +3 i.p. twice a day (once at day 0); 3) −60 min i.p. and +10 i.p. Statistical significance (*p*) was evaluated by the Student's *t*-test. Lethality of the LPS-treated group is indicated in brackets. ST 261 = propionyl-L-carnitine chloride; ST 780 = propionyl-L-carnitine chloride ester with β-hydroxybutyric acid; ST 880 = propionyl-L-carnitine chloride ester with γ-hydroxybutyric acid; ST 921 = propionyl-L-carnitine chloride amide with arginine hydrochloride.

and ST 878 significantly reduced ($p < 0.05$) TNF levels. No differences in serum IL-6 levels were found in mice intoxicated with LPS and treated with ST 284 and ST 878. The survival of LPS-challenged mice was not improved following administration of any of these compounds (Table 5).

*Isovaleryl-L-carnitine and six derivatives:* Among the compounds belonging to this class, only two derivatives, namely ST 687 and ST 1037, were effective in significantly reducing ($p < 0.05$) serum TNF levels (Table 6). Even if none of the investigated compounds significantly improved the survival of the LPS-challenged mice, isovaleryl-L-carnitine (ST 551) administration still allowed a better recovery of health conditions of survived mice. The class distributions of the animal health state, based on animal body temperature and evaluated at different days after treatment, are shown in Fig. 3.

### Discussion

Septic shock is an increasingly serious health problem in hospitals, especially in intensive care units, despite the use of multiple antibiotics, surgical drainage, and intervention including vasopressor and metabolic support. It was estimated that this disease causes 100 000 deaths annually in the United States.[23] It is generally accepted that the primary mediator of the pathophysiological changes occurring in Gram-negative sepsis is LPS, which in turn stimulates host macrophages to produce a cascade of endogenous mediators responsible for many alterations in host physiology. There are many reports showing that

**Table 5.** Effects of isobutyryl-L-carnitine (ST 284) and its derivatives (ST 730, ST 878) on both serum TNF and IL-6 production, and lethality of mice challenged with LPS

| | | TNF | | | IL-6 | | | Lethality |
|---|---|---|---|---|---|---|---|---|
| Compound | Treatment | % variation | No. of mice | *p* | % variation | No. of mice | *p* | Dead/total |
| ST 284 | *1* (50 mg/kg) | −6 | 16 | n.s. | +12 | 8 | n.s. | 6/8 (3/8) |
| ST 730 | *1* (50 mg/kg) | −51 | 7 | <0.05 | | | | |
| | *2* (50 mg/kg) | – | – | – | – | – | – | 5/8 (6/8) |
| ST 878 | *1* (50 mg/kg) | −51 | 8 | <0.05 | −23 | 8 | | |
| | *2* (50 mg/kg) | – | – | – | – | – | – | 2/8 (3/8) |

Treatments were performed by injecting LPS 30 mg/kg i.p. at time zero and administering the compounds, at the dose indicated, as follows: *1*) −60 min i.p. and +10 min i.v., *2*) from day −1 to day +3 i.p. twice a day (once at day 0). Statistical significance (*p*) was evaluated by the Student's *t*-test. Lethality of the LPS-treated group is indicated in brackets. ST 284 = isobutyryl-L-carnitine chloride; ST 730 = isobutyryl-L-carnitine chloride ester with β-hydroxybutyric acid; ST 878 = isobutyryl-L-carnitine ester with γ-hydroxybutyric acid.

**Table 6.** Effects of isovaleryl-L-carnitine chloride (ST 551) and its derivative (ST 743, ST 687, ST 994, ST 803, ST 899, ST 1037) on serum TNF production, and lethality of mice challenged with LPS

| Compound | Treatment | TNF % variation | TNF No. of mice | TNF $p$ | Lethality Dead/total |
|---|---|---|---|---|---|
| ST 551 | *1* (50 mg/kg) | −25 | 16 | n.s. | 11/24 (14/24) |
| ST 743 | *1* (50 mg/kg) | −23 | 8 | n.s. | 6/8 (6/8) |
| ST 687 | *1* (50 mg/kg) | −44 | 8 | <0.05 | 5/8 (7/8) |
| | *2* (200 mg/kg) | – | – | – | 8/8 (4/8) |
| ST 944 | *1* (50 mg/kg) | +29 | 8 | n.s. | 5/8 (7/8) |
| ST 803 | *1* (5 mg/kg i.p. and *1* mg/kg i.v.) | +16 | 8 | n.s. | 8/8 (4/8) |
| | *4* (5 mg/kg) | – | – | – | 3/5 (5/7) |
| ST 899 | *1* (5 mg/kg) | −27 | 8 | n.s. | 6/8 (6/8) |
| ST 1037 | *1* (5 mg/kg i.p. and *1* mg/kg i.v.) | −44 | 8 | <0.05 | 5/8 (5/8) |

Treatments were performed by injecting LPS 30 mg/kg i.p. at time zero and administering the compounds, at the dose indicated, as follows: *1*) −60 min i.p. and +10 min i.v., *2*) from day −1 to day +3 i.p. twice a day (once at day 0); 4) −60 min i.p. Statistical significance ($p$) was evaluated by the Student's $t$-test. Lethality of the LPS-treated group is indicated in brackets. ST 551 = isovaleryl-L-carnitine chloride; ST 743 = isovaleryl-L-carnitine tartrate acid ester with γ-hydroxybutyric acid; ST 803 = isovaleryl-L-carnitine bromide ester with *Z*-3-(6-chloro)-phthalidiliden-ethanol; ST 899 = isovaleryl-L-carnitine bromide ester with *Z*-3-(5-chloro)phthalidiliden-ethanol; ST 1037 = isovaleryl-L-carnitine chloride ester with dodecanol.

over-production of several cytokines such as IL-1, IL-6, TNF, and IFN-γ is associated with severe sepsis. Among these cytokines, however, TNF has been shown to play a key role in many metabolic derangements occurring during septic shock,[24] and its abnormally high serum levels are often correlated with poor prognosis.[25,26] Moreover, TNF can also be found in the serum of animals experimentally intoxicated with LPS and additionally causes, when injected in animals, a toxic syndrome indistinguishable from endotoxaemia.[27–29]

Experimentally induced endotoxin shock in laboratory animals, although it does not exactly reproduce all the alterations of septic shock, has become a valuable and very convenient model for studying sepsis. In fact, it reliably mimics many physiological as well as immunological dysregulations occurring in this disease.[24]

By using this model, the authors found that LPS injection in mice caused a dramatic reduction in body temperature. This decrease was evident no sooner than 24 h following LPS administration and

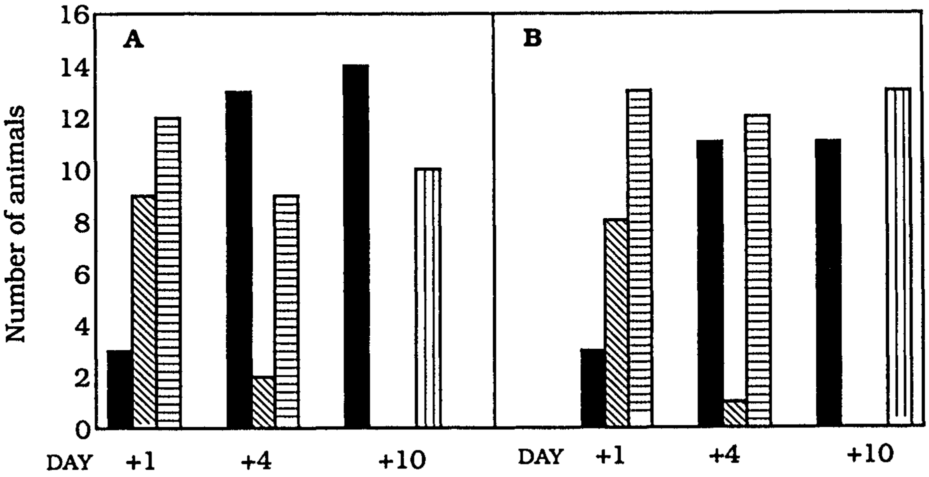

FIG. 3. Health conditions, as evaluated by body temperature, at days +1, +4 and +10 of LPS-injected mice treated either with saline (panel A) or with isovaleryl-L-carnitine (panel B) according to the treatment protocol reported in Table 2. ■, Dead; ▧, Very ill (<32°C); ▤, Fair (32–35°C); ▥, Survivors.

lasted for two more days. During this time, the LPS-injected mice were very sick, and they started to recover only from the fourth day after LPS challenge.

At variance with this finding, other investigators reported an increase in body temperature in different animal models[30,31] as well as in humans[32] following LPS injection. In agreement with our results, however, Pogrebniak *et al.*[33] found that C3H/HeN mice injected with LPS (30 mg/kg, i.p.) were markedly hypothermic. Additionally, Handley *et al.*[34] made similar observations in Balb/c mice injected i.v. with 10 mg/kg LPS. From these studies, it can be argued that hypothermia is a typical physiological response of mice to LPS and appears to be independent of the strain used. In the present model, moreover, smaller decreases in body temperature were observed when lower doses of LPS (down to 0.3 mg/kg) were employed, while a dose as low as 0.03 mg/kg was completely ineffective (data not shown). Furthermore, the decrease in body temperature was paralleled by a concomitant body weight loss, which appeared to be related to the dose of LPS (data not shown). The maximum decrease of body weight was observed 48 h after LPS challenge, then the mice slowly began to recover their normal weight. Taken together, these data indicate that body temperature and body weight are valuable parameters to monitor health conditions of mice during endotoxaemia. In a preliminary experiment, performed to ascertain the time course of TNF production, it was found that serum TNF peaked as early as 1 h following LPS injection, and became undetectable by 4 h. This result is consistent with similar studies reported by other investigators in different animal models[35–37] and in humans.[32]

In this experimental model L-carnitine and some acyl derivatives were tested for possible protective effects in LPS-challenged mice. A role of L-carnitine in the treatment of sepsis was first suggested by Border *et al.*,[38] who hypothesized that some septic processes might be associated with an impairment of lipid oxidation due to systemic depletion of L-carnitine.[38] Support to this hypothesis was lent by different investigators, who observed a significant improvement in survival of LPS-challenged animals following treatment with L-carnitine.[39,40]

In this LPS shock model, L-carnitine and its derivative ST 784 were able to reduce serum TNF levels, although this decrease was not always statistically significant. Similarly, the compounds derived from propionyl-L-carnitine and isobutyryl-L-carnitine were effective in reducing serum TNF release in LPS-challenged mice. In contrast, only two isovaleryl-L-carnitine derivatives, namely ST 687 and ST 1037, were found to significantly decrease TNF levels. However, L-carnitine and isovaleryl-L-carnitine, which differently affected serum TNF levels, were both able to improve health conditions of LPS-injected mice and to lightly reduce lethality as well. Different treatment schedules and different dosages may be necessary to ascertain the real therapeutic potential of these compounds in counteracting LPS intoxication.

The authors found that serum TNF and IL-6 levels were modulated differently by treatment with L-carnitines. In fact, in most cases where serum TNF was significantly decreased, concomitant significantly high levels of serum IL-6 were observed. This is of interest in the light of the suggestion that IL-6 may act as a negative modulator of TNF production.[30,41] In addition, IL-6 was found to induce hyporesponsiveness to endotoxin in mice,[42] and to inhibit the release of TNF in mice as well as in human U937 cells and monocytes *in vitro*.[43] Furthermore, it is known that TNF and IL-6 synthesis is regulated differently by various agents. High c-AMP levels, for example, were reported to inhibit TNF synthesis while inducing IL-6 production.[44–45] Additionally, Marcinkiewicz[46] observed that the production of these cytokines by murine peritoneal macrophages was affected differently by prostaglandins $PGE_2$ and $PGI_2$. In fact, while both prostaglandins inhibited the release of TNF, they increased IL-6 production at the same time.

It would be tempting to speculate, therefore, that L-carnitines might increase prostaglandin synthesis and c-AMP levels, the latter either directly or via prostaglandins, thereby decreasing TNF synthesis and increasing IL-6 levels. IL-6 in turn would further dampen the production of TNF.[30,41] Although additional investigations are needed, reports showing that L-carnitine and some of its congeners were able to positively affect prostaglandins release from macrophages[47] support this hypothesis.

Although TNF release has been reported to be a prominent event during endotoxaemia,[2,5,36,48] the authors' results also point out that TNF by itself cannot be the only crucial mediator of lethality. In fact, it was found that the significant reductions in TNF levels brought about by several L-carnitine derivatives were not correlated with a significant reduction of lethality, though prolongation of mean survival time (data not shown) and ameliorated health conditions of treated animals were observed. The involvement of other critical factors in addition to TNF is supported by several studies showing that TNF by itself is necessary, although not sufficient, to cause lethality during endotoxaemia[4,8,49,50]

Even though many questions concerning the ultimate molecular mechanism of action of L-carnitines in endotoxaemia are yet to be answered, it is envisaged that these compounds may be helpful, when associated with conventional therapy, in that they can effectively reduce TNF levels and ameliorate the host's metabolic processes. In conclusion, these results would justify clinical trials with L-carnitine in the treatment of sepsis. Indeed, preliminary reports[12,51] indicate that early supplementation of L-carnitine in the therapy of septicaemia appears to counteract the onset of the metabolic cascade leading to septic shock.

### References

1. Parrillo JE. Septic shock in humans: advances in the understanding of pathogenesis, cardiovascular dysfunction, and therapy. *Ann Intern Med* 1990; **113**: 227–242.
2. Glauser MP, Zanetti G, Baumgartner JD, Cohen J. Septic shock: pathogenesis. *Lancet* 1991; **338**: 732–736.
3. Dunn DL. Role of endotoxin and host cytokines in septic shock. *Chest* 1991; **100**: 164S–168S.
4. Tracey KJ, Fong Y, Hesse DG, *et al.* Anti-cachectin/TNF monoclonal antibodies prevent septic shock during lethal bacteremia. *Nature* 1987; **330**: 662–664.
5. Beutler B, Cerami A. The endogenous mediator of endotoxic shock. *Clin Res* 1987; **35**: 192–197.
6. Cerami A, Ikeda Y, Le Trang N, Hotez PJ, Beutler B. Weight loss associated with an endotoxin-induced mediator from peritoneal macrophages: the role of cachectin (tumour necrosis factor). *Immunol Lett* 1985; **11**: 173–177.
7. Klosterhalfen B, Horstmann-Jungemann K, Vogel P, *et al.* Time course of various inflammatory mediators during recurrent endotoxemia. *Biochem Pharmacol* 1992; **43**: 2103–2109.
8. Calandra T, Baumgartner JD, Grau GE, *et al.* Prognostic values of tumor necrosis factor/cachectin, IL-1, IFNα and IFNγ in the serum of patients with septic shock. *J Infect Dis* 1990; **161**: 982–987.
9. Fong Y, Tracey, KJ, Moldawer LL, *et al.* Antibodies to cachectin/TNF reduce IL-1β and IL-6 appearance during lethal bacteraemia. *J Exp Med* 1989; **170**: 1627–1633.
10. Shaunak S, Wendon J, Monteil M, Gordon AM. Septic scarlet fever due to *Streptococcus pyogenes* cellulitis. *Q J Med* 1988; **69**: 921–925.
11. Marks JD, Marks CD, Luce JM. Plasma tumour necrosis factor in patients with septic shock. Mortality rate, incidence of adult respiratory distress syndrome and effects of methyl-prednisolone administration. *Am Rev Respir Dis* 1990; **141**: 94–97.
12. Nanni G, Pittiruti M, Giovannini I, Boldrini G, Ronconi P, Castagneto M. Plasma carnitine levels and urinary excretion during sepsis. *J Parenter Enteral Nutr* 1985; **9**: 483–490.
13. Kirijama M. Effect of L-carnitine administration on lipid utilization in sepsis. *J Juzen Med Soc* 1986; **95**: 619–632.
14. Davis TA, Crady SK, Strong SA, *et al.* Increased acylcarnitine clearance and excretion in septic rats. *Biomed Biochim Acta* 1991; **1**: 81–86.
15. Bremer J. Carnitine. Metabolism and function. *Physiol Rev* 1983; **63**: 1420–1480.
16. Schinetti ML, Mazzini A. Effect of L-carnitine on human neutrophil activity. *Int J Tiss Reac* 1986; **8**: 199–203.
17. Fieren MWJA, Bemd van den, GJCM, Ben-Efraim S, Bonta IL. Prostaglandin E2 inhibits the release of tumour necrosis factor-α rather than interleukin 1β from human macrophages. *Immunol Lett* 1991; **31**: 85–90.
18. Strieter RM, Remick DG, Ward PA, *et al.* Cellular and molecular regulation of tumour necrosis factor-alpha production by pentoxifylline. *Biochem Biophys Res Commun* 1988; **155**: 1230–1236.
19. Waage A, Brandtzaeg P, Halstensen A, Kierulf P, Espevik T. The complex pattern of cytokines in serum from patients with meningococcal septic shock. *J Exp Med* 1989; **169**: 333–338.
20. Kelly NM, Cross AS. Interleukin 6 is a better marker of lethality than tumour necrosis factor in endotoxin treated mice. *FEMS Microbial Immunol* 1992; **89**: 317–322.
21. Aarden L, DeGroot ER, Schaap OL, Lansdorp PM. Production of hybridoma growth factor by human monocytes. *Eur J Immunol* 1987; **17**: 1411–1416.
22. Flick DA, Gifford GE. Comparison of *in vitro* cell cytotoxic assays for tumour necrosis factor. *J Immunol Meth* 1984; **68**: 167–175.
23. Cohen J, Glauser MP. Septic shock: treatment. *Lancet* 1991; **338**: 736–739.
24. Starnes HF, Warren RS, Jeevanandam M, *et al.* Tumour necrosis factor and the acute metabolic response to tissue injury in man. *J Clin Invest* 1988; **82**: 1321.
25. Dames P, Reuter A, Gysen P, Demonty J, Lamy M, Franchimont P. Tumour necrosis factor and interleukin-1 serum levels during severe sepsis in humans. *Crit Care Med* 1989; **17**: 975–978.
26. Debets JMH, Kampmeijer R, van der Linden MPMH, Buurman WA, van der Linden CJ. Plasma tumour necrosis factor and mortality in critically ill septic patients. *Crit Care Med* 1989; **17**: 489–494.
27. Natanson C, Eichenhols PW, Danner RL. Endotoxin and tumour necrosis factor challenges in dogs simulate the cardiovascular profile of human septic shock. *J Exp Med* 1989; **169**: 823–832.
28. Beutler B, Milsark IW, Cerami A. Passive immunization against cachectin/tumour necrosis factor protects mice from lethal effect of endotoxin. *Science* 1985; **229**: 869–871.
29. Vassalli P. The pathophysiology of tumour necrosis factors. *Annu Rev Immunol* 1992; **10**: 411–452.
30. Flohé S, Heinrich PC, Schneider J, Wendel A, Flohé L. Time course of IL-6 and TNFα release during endotoxin-induced endotoxin tolerance in rats. *Biochem Pharmacol* 1991; **41**: 1607–1614.
31. Mozes T, Ben-Efraim S, Tak CJAM, Heiligers JPC, Saxena PR, Bonta IL. Serum levels of tumour necrosis factor determine the fatal or non fatal course of endotoxic shock. *Immunol Lett* 1991; **27**: 157–162.
32. Michie HR, Manogue KR, Spriggs DR, *et al.* Detection of circulating tumour necrosis factor after endotoxin administration. *N Engl J Med* 1988; **318**: 1481–1486.
33. Pogrebniak HW, Merino MJ, Hahn SM, Mitchell JB, Pass HI. Spin trap salvage from endotoxaemia—the role of cytokine down-regulation. *Surgery* 1992; **112**: 130–139.
34. Handley DA, Van Valen RG, Melden MK, Houlihan WJ, Saunders RN. Biological effects of the orally active platelet activating factor receptor antagonist SDZ 64-412. *J Pharmacol Exp Ther* 1988; **247**: 617–623.
35. Niehorster M, Schonharting M, Wendel A. A novel xanthine derivative counteracting *in vivo* tumour necrosis factor-alpha toxicity in mice. *Circulatory shock* 1992; **37**: 270–273.
36. Smith III EF, Slivjak MJ, Bartus O, Esser KM. SK&F 86002 inhibits tumour necrosis factor formation and improves survival in endotoxaemic rats. *J Cardiovasc Pharmacol* 1991; **18**: 721–728.
37. Weinberg JR, Boyle P, Meager A, Guz A. Lipopolysaccharide, tumour necrosis factor, and interleukin-1 interact to cause hypotension. *J Lab Clin Med* 1992; **120**: 205–211.
38. Border JR, Burns GP, Rumph C, Shenk WGjr. Carnitine levels in severe infection and starvation: a possible key to the prolonged catabolic state. *Surgery* 1970; **68**: 175–179.
39. Takeyama N, Takagi D, Matsuo N, Kitazawa Y, Tanaka T. Altered hepatic fatty acid metabolism in endotoxicosis: effect of L-carnitine on survival. *Am J Physiol* 1989; **256**: E31-E38.
40. Yoshitake J, Nomoto Y, Kono S, *et al.* Metabolic deterioration in shock state and its modulation. In: *Molecular and cellular aspects of shock and trauma.* New York: Alan R. Liss, 1983: 21–38.
41. Rola-Pleszczynski M, Stankova J. Cytokine gene regulation by $PGE_2$, $LTB_4$ and PAF. *Mediat Inflamma* 1992; **1**: 5–8.
42. Van der Meer JWM, Helle M, Aarden A. Comparison of the effect of recombinant interleukin 6 and recombinant interleukin 1 on nonspecific resistance to infection. *Eur J Immunol* 1989; **19**: 413–416.
43. Aderka D, Le J, Vilcek J. IL-6 inhibits lipopolysaccharide-induced tumour necrosis factor production in cultured human monocytes, U937 cells, and in mice. *J Immunol* 1989; **143**: 3517–3523.
44. Katakami Y, Nakao Y, Koizumi NT, Katakami N, Ogawa R, Fujita T. Regulation of tumour necrosis factor production by mouse peritoneal macrophages: the role of cellular cyclic AMP. *Immunology* 1988; **64**: 719–724.
45. Zhang Y, Lin JX, Vilcek J. Synthesis of interleukin 6 (interferon-$beta_2$/B cell stimulatory factor 2) in human fibroblasts is triggered by an increase in intracellular cyclic AMP. *J Biol Chem* 1988; **263**: 6177–6182.
46. Marcinkiewicz J. *In vitro* cytokine release by activated murine peritoneal macrophages: role of prostaglandins in the differential regulation of tumour necrosis factor alpha, interleukin 1, and interleukin 6. *Cytokine* 1991; **3**: 327–332.
47. Elliott GR, Lauwen APM, Bonta IL. The effect of acute feeding of carnitine and propionyl carnitine on basal and A23187-stimulated eicosanoid release from rat carrageenin-elicited peritoneal macrophages. *Br J Nutr* 1990; **64**: 497–503.
48. Girardin E, Grau GE, Dayer JM, *et al.* Tumour necrosis factor and interleukin-1 in the serum of children with severe infectious purpura. *N Engl J Med* 1988; **319**: 397–400.
49. Franks AK, Kujawa KI, Yaffe LJ. Experimental elimination of tumour necrosis factor in low-dose endotoxin models has variable effects on survival. *Infection and Immunity* 1991; **59**: 2609–2614.
50. Feuerstein G, Hallenbeck JM, Vanatta B, Rabinovici R, Perera PY, Vogel SN. Effect of Gram-negative endotoxin on levels of serum corticosterone, TNFα, circulating blood cells, and the survival of rats. *Circ Shock* 1990; **30**: 265–278.
51. Scaglione F, Cogo R, Mascola S, Fraschini F. Use of L-carnitine in patients affected by sepsis. *Argomenti di Chemioantibiotico Terapia* 1992; **2**: 210–215.

ACKNOWLEDGEMENTS. Dr F. Beltrami is gratefully acknowledged for artwork and for editing this manuscript. The excellent technical assistance of Mrs C. Chiapparino, Mrs S. Manganello, Mrs M. L. Pacello, Mrs V. Parrella, Ms B. Leoni, Mr A. Marconi and Mr A. Rosi is also acknowledged.
